# A Self-Alignment Algorithm for SINS Based on Gravitational Apparent Motion and Sensor Data Denoising

**DOI:** 10.3390/s150509827

**Published:** 2015-04-27

**Authors:** Yiting Liu, Xiaosu Xu, Xixiang Liu, Yiqing Yao, Liang Wu, Jin Sun

**Affiliations:** Key Laboratory of Micro-Inertial Instrument and Advanced Navigation Technology, Ministry of Education, School of Instrument Science and Engineering, Southeast University, Nanjing 210096, China, E-Mails: gcdlyt1985@163.com (Y.L.); scliuseu@163.com (X.L.); yucia@sina.com (Y.Y.); wuliang200809@126.com (L.W.); sunjin8607986@126.com (J.S.)

**Keywords:** strapdown inertial navigation (SINS), self-alignment, gravitational apparent motion, denoising

## Abstract

Initial alignment is always a key topic and difficult to achieve in an inertial navigation system (INS). In this paper a novel self-initial alignment algorithm is proposed using gravitational apparent motion vectors at three different moments and vector-operation. Simulation and analysis showed that this method easily suffers from the random noise contained in accelerometer measurements which are used to construct apparent motion directly. Aiming to resolve this problem, an online sensor data denoising method based on a Kalman filter is proposed and a novel reconstruction method for apparent motion is designed to avoid the collinearity among vectors participating in the alignment solution. Simulation, turntable tests and vehicle tests indicate that the proposed alignment algorithm can fulfill initial alignment of strapdown INS (SINS) under both static and swinging conditions. The accuracy can either reach or approach the theoretical values determined by sensor precision under static or swinging conditions.

## 1. Introduction

Initial alignment is always a precondition for an Inertial Navigation System (INS) to navigate [1,2]. For INS, initial alignment is the acquisition of the initial velocity, position and attitude. Because the velocity and position are easy to be obtained by an external reference system, such as the Global Positioning System (GPS), initial alignment mainly refers to the acquisition of initial attitude. Strapdown INS (SINS) uses a mathematical platform as a navigation platform, where the initial alignment specifically obtains the matrix between the body frame and navigation frame [3,4,5]. In SINS, the Inertial Measurement Unit (IMU) is directly installed in the vehicle, which easily suffers from the disturbances caused by running conditions, therefore making achievement of a robust alignment algorithm difficult.

The technical manuals of the Octans system developed by the IxBlue Company (Marly le Roi, France) claim that the Octans can complete initial alignment under any swinging conditions within 5 min by observing the drift of gravity in an inertial frame (gravitational apparent motion) [6,7]. Protection of the technology and commercial interests may be the reasons why no details about the realization method are given in these manuals. Inspired by the alignment idea of tracing apparent motion, many realization methods have been proposed since 2000, which can be divided into two types, namely attitude determination based on dual vectors and vector-operational [8,9,10,11,12,13,14,15,16,17,18,19,20].

Both methods obtain initial attitude with non-collinear vectors of gravitational apparent motions which are formed by projecting accelerometer measurements into an inertial frame [10,11,12], but in the former one, the initial alignment problem involves solving the matrix between the initial body frame and initial navigation frame; while in the latter, this problem becomes how to solve the matrix between the initial body frame and the current navigation frame. Previous analyses [9,21,22,23] indicate that when we use these methods under static conditions, accurate position information is necessary in the former case, because the projection of the theoretical gravity in the initial navigation frame is needed, while in the latter no external information is needed, which means that it is a complete self-alignment method.

**Table 1:** The parameters of the gyros and accelerometers.

**Gyros**			
Constant bias	＜0.01 ^○^/h (1 *δ*)	Nonlinearity of scale factor	≤50 ppm (1 *δ*)
Repetitiveness of constant bias	＜0.01 ^○^/h (1 *δ*)	Repetitiveness of scale factor	≤50 ppm (1 *δ*)
Random walk	$<0.005{\;}^{\circ }/\sqrt{\text{h}}$	Measuring range	−300～+300 ^○^/s
**Accelerometers**			
Measuring range	−20～+20 g	bias	＜5 × 10^−5^ g
Threshold	＜5 × 10^−6^ g	Temperature coefficient of bias	＜6 × 10^−5^/^○^C (−40～+40 ^○^C)
Repetitiveness of scale factor	＜3.5 × 10^−5^ (1δ)	Repetitiveness of bias	＜2.5 × 10^−5^ (1δ)
Temperature coefficient of scale factor	＜6 × 10^−5^/^○^C (−40～+40 ^○^C)	bandwidth	>800 Hz

**Figure 1:**
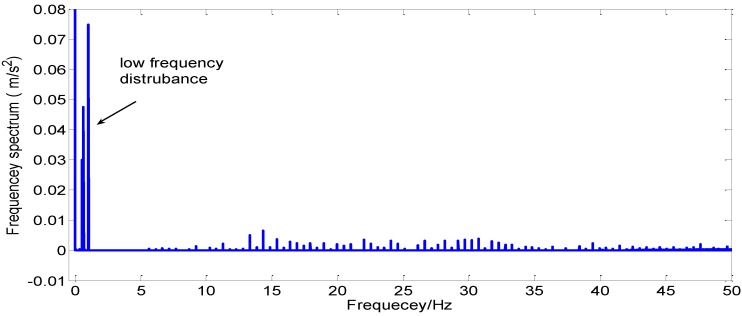
The z axis acceleration frequency spectrum under mooring conditions.

However, both of these methods easily suffer from the random noise contained in the accelerometer measurements and because of that the measured acceleration are used to construct the apparent motion directly [13,14,15,16,17,18,19,20]. Apart from random noise, many other errors also exist in the measurements such as random walk, nonlinearity of scale factors and so on. The details of the gyros and the accelerometers which are used in this paper are shown in Table 1. Figure 1 shows the frequency spectrum of the z**-**axis acceleration of an IMU installed on a turntable, when the turntable is swinging to simulate a ship under mooring conditions. The sampling frequency is 200 Hz. It can be concluded that the accelerometer measurement not only contains the intrinsic sensor noise but also contains some low-frequency and high-frequency disturbance**s**. All of these errors have an effect on the accuracy of the initial alignment.

In order to realize the alignment under mooring conditions, the intrinsic sensor noise, low frequency and the high-frequency disturbances should be eliminated. Fortunately, the influence of fixed low frequency disturbances caused by the seawaves can be attenuated by the dual vector and vector-operation alignment method**s** and for that the inertial navigation frame is specially selected. The intrinsic sensor noise and the external random noise should be eliminated as much as possible [13].

To solve this problem [14,17] integrated an apparent motion to form an apparent velocity taking advantage of the smoothing effects of integration. Furthermore [15,16] introduced the wavelet denoising technique to eliminate the sensor noise and [17,18,19,20] introduced a low-pass filter to remove the noise taking advantage of the different characteristics of noise and apparent motion. In [9,22,23] the authors designed a parameter recognition algorithm based on the recursive least square (RLS) algorithm in which they reconstruct the theoretical apparent motion from the calculated apparent motion that does not contain random noise. In [24,25,26] the authors combined a Kalman filter and a IIR filter to reduce the external disturbance and the sensor noise. Although all methods seem to be suitable for the studied objects, it cannot be neglected that any integration method extends the alignment time making it costly. It is also difficult to find universal low-pass filter parameters applicable for the complex noise and dynamic environment. Meanwhile, the time-delay problem should be further studied because SINS is a real-time system [19,20]. Similarly, with RLS, optimal parameters can be acquired when minimizing the errors between the fitted data and measured data but it is vulnerable to wild values [9,22,23], and the wavelet denoising technique is always used in a post-processing case for the tremendous computing workload. 

In this paper, the alignment problem for the vehicle with swinging but no translational motion is studied. The alignment based on vector-operation is selected in order to reduce the alignment time and improve the accuracy of the alignment. To reduce the influence of the random noise of measurement of IMU, an online parameter-adjusted Kalman filter technology is introduced to estimate the useful signals based on the knowledge about the system. The online parameter-adjusted Kalman filter technology can adjust the gain matrix and the measurement noise variance matrix according to the motion of the vehicle, which causes little time delay. Combining the alignment method based on tracing apparent motion and vector-operation with denoising method based on the online parameter-adjusted Kalman filter revealed exciting results in simulations, *i.e.*, turntable tests and vehicle tests.

The rest of this paper is organized as follow: in Section 2, the gravitational apparent motion is introduced and the alignment algorithm is designed and analyzed with a simulation. In Section 3 an online filtering technology is introduced to ease the negative effects of the random measurement noise and a new reconstruction algorithm of the gravitational apparent motion is proposed. In Section 4, turntable tests and vehicle tests are carried out to verify the effectiveness of this novel algorithm. Finally, the paper’s findings are summarized in Section 5.

## 2. The Gravitational Apparent Motion and a Novel SINS Alignment Method

### 2.1. Self-Alignment Method Based on Gravitational Apparent Motion and Vector-Operation

The SINS alignment is to obtain the initial attitude matrix $C_{b}^{n}(t)$ between the current body frame and the current navigation frame. In the self-alignment method based on the gravitational apparent motion and vector-operation, $C_{b}^{n}(t)$ can be decomposed into two parts which are [22]:

(1) $$C_{b}^{n}(t)=C_{i_{b0}}^{n}(t)C_{b}^{i_{b0}}(t)$$

where *b* and *n* denote body frame and navigation frame respectively; $i_{b0}$ is the inertial frame formed by fixing initial body frame in inertial space; $C_{N}^{M}$ is the attitude matrix between frame *M* and frame *N*.

The matrix $C_{b}^{i_{b_{0}}}(t)$ in Equation (1) can be updated with the measurements of gyros as follows:

(2) C^·ib0b(t)=C^ib0b(t)(ω˜ibb×)=C^ib0b(t)(ω˜ib0bb×)

where the superscripts “︿” and “～” denote calculated value and measurement value, respectively; ${\tilde{\omega }}_{i_{b0}b}^{b}$ is the gyro measurement value.

According to Equations (1) and (2), solving $C_{n}^{b}(t)$ in SINS alignment is thus converted into solving the matrix between the inertial frame and the current navigation frame, $C_{n}^{i_{b_{0}}}(t)$.

#### 2.1.1. Gravitational Apparent Motion

**Figure 2:**
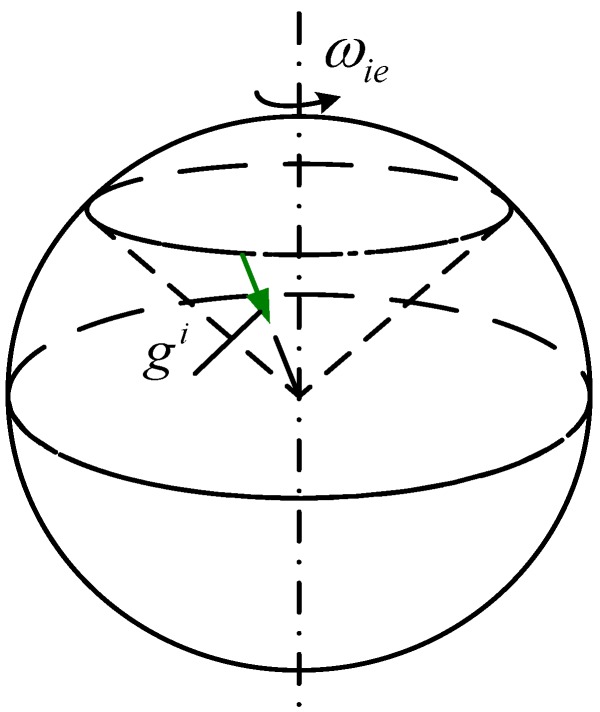
Trajectory of the gravitational apparent motion in inertial space.

The concept of apparent motion in INS is initially used to describe the characteristics of gyros. Researchers generally observe that gyros that are stable in the inertial frame, but in the navigation frame, the gyros and the inertial frame revolve around the Earth. The gravitational apparent motion is usually defined as the track of the gravity vector, which is stable in the Earth and rotating in the inertial frame. According to [7,8], the gravitational apparent motion in inertial frame is shown in Figure 2. In inertial frame, gravitational apparent motion is illustrated as a cone with the vertex at the Earth center and the cone axis coinciding with Earth’s rotating axis, the conical bottom radius is determined by the latitude where the vehicle is located. Notably, the formation of this cone is independent of the selection of inertial frame, but the specific mathematical expressions are related to the selection [9,22]. 

#### 2.1.2. Alignment Method Based on Gravitational Apparent Motion

**Figure 3:**
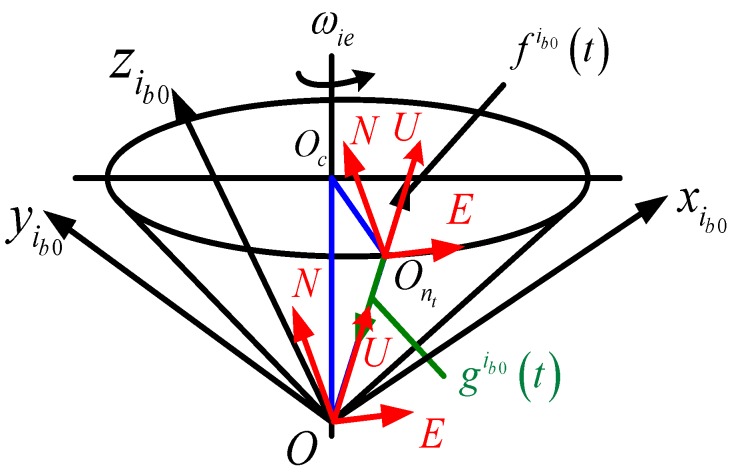
Alignment mechanism based on gravitational apparent motion.

The analysis method from [9,22] where “East-North-Up (ENU)” is selected as the navigation frame, the geographical relationship between navigation frame and the cone of apparent motion can be successfully analyzed. As shown in Figure 3, the points of O and $O_{c}$ denote the vertex (the Earth’s center) and conical bottom center, respectively. At the moment *t*, the origin of frame *n* is located at $O_{n_{t}}$ which is at the circle of conical bottom. The vector ${\vec{OO}}_{n}$ connecting the point O and $O_{n}$ is consistent with the Up-axis of frame, but it is reverse from the direction of $g^{i}(t)$. The vector ${\vec{OO}}_{c}$ connecting the point O and $O_{c}$ is consistent with the Earth rotating axis. The product of ${\vec{OO}}_{c}\times {\vec{OO}}_{n}$ is consistent with the east-axis of n, while that of U×E is consistent with the north-axis of frame *n*. When the projections of all axes in navigation frame are determined in the inertial frame, the matrix ${\text{C}}_{n}^{i_{b_{0}}}(t)$ can be constructed as follows [22]:

(3) $$C_{i_{b0}}^{n}(t)={\left[\begin{array}{ccc} E^{T} & N^{T} & U^{T} \end{array}\right]}^{T}$$

where ***E***, ***N***, ***U*** are the projections of the axes of frame *n* in frame $i_{b0}$.

**Figure 4:**
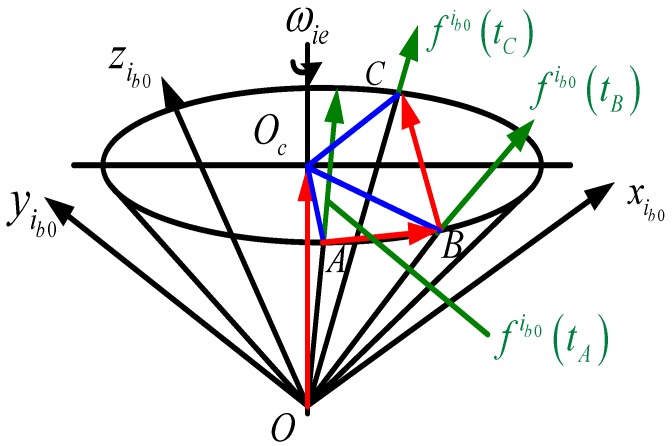
Calculation method for cone axis.

According to the above analysis, the initial alignment for SINS can be transformed into acquiring the projections of the navigation frame axes into the inertial frame. There are two problems which should be resolved, *i.e.*, how to acquire gravitational apparent motion in frame $i_{b0}$ and how to construct the vectors in the apparent motion cone.

In order to that we define “Right-Forward-Up” as the body frame, and assume that the axes of the IMU frame coincide with those body frames. Then, the gravitational apparent motion in inertial frame can be calculated as follows:

(4) $${\hat{f}}^{i_{b0}}(t)=C_{b}^{i_{b0}}(t){\tilde{f}}^{b}(t)$$

where, ${\tilde{f}}^{b}(t)$ is the accelerometer measurement in frame *b*. According to definitions of navigation frame and body frame, as well as the vector relationship described in Figure 3, there is $f^{i_{b_{0}}}(t)=-g^{i_{b_{0}}}(t)$. Here, the first problem is solved.

Because the vector ${\vec{OO}}_{n}$ coincides with the Up-axis of navigation frame, the vector ***U*** can be directly calculated as follows:

(5) U^=OO→n=‒g^n||g^n||=C^ib0nf^ib0|C^ib0nf^ib0|=f^ib0||f^ib0||

Therefore, the precondition of solving the vector ***E*** is to solve the vector ${\vec{OO}}_{c}$. As described in the above sections, the cone axis is the Earth’s rotation axis which has a fixed direction. Then, ${\hat{f}}^{i_{b_{0}}}$ at three different moments can be selected to construct ${\vec{OO}}_{c}$ through vector-operations. As shown in Figure 4, the apparent motion vectors ${\hat{f}}^{i_{b_{0}}}(t_{A})$, ${\hat{f}}^{i_{b_{0}}}(t_{B})$ and ${\hat{f}}^{i_{b_{0}}}(t_{C})$ at three different moments *t*_A_, *t*_B_ and *t*_C_ are used to construct procedure vectors $\vec{AB}={\hat{f}}^{i_{b_{0}}}(t_{B})-{\hat{f}}^{i_{b_{0}}}(t_{A})$ and $\vec{BC}={\hat{f}}^{i_{b_{0}}}(t_{C})-{\hat{f}}^{i_{b_{0}}}(t_{B})$. Thus, the vector ${\vec{OO}}_{c}$ can be constructed as follows:

(6) $${\vec{OO}}_{c}=\frac{\vec{AB}\times \vec{BC}}{\left\|\vec{AB}\times \vec{BC}\right\|}$$

With the vector ${\vec{OO}}_{c}$ and ***U***, the vector ***E*** can be constructed as follows:

(7) $$\hat{E}=\frac{{\vec{OO}}_{c}\times \hat{U}}{\left\|{\vec{OO}}_{c}\times \hat{U}\right\|}$$

Furthermore, the vector ***N*** can be constructed as follows:

(8) $$\hat{N}=\frac{\hat{U}\times \hat{E}}{\left\|\hat{U}\times \hat{E}\right\|}$$

According to equations from Equations (4)–(8), ${\text{C}}_{n}^{i_{b_{0}}}(t)$ in Equation (3) can be constructed as follows:

(9) $${\text{C}}_{i_{b_{0}}}^{n}(t)={\left[\begin{array}{ccc} {\hat{E}}^{T} & {\hat{N}}^{T} & {\hat{U}}^{T} \end{array}\right]}^{T}$$

Based on Equations (1), (2) and (9), initial alignment for SINS can be fulfilled, and the theoretical alignment accuracy of this method can be expressed as follows:

(10) $$\phi_{x}=\frac{\nabla_{N}}{g},\phi_{y}=-\frac{\nabla_{E}}{g},\phi_{z}=\frac{\varepsilon_{E}}{\omega_{ie}\cos L}$$

where, $\nabla_{E}$ and $\nabla_{N}$ are the equivalent accelerometer errors of the east and north respectively; $\varepsilon_{N}$ is the equivalent gyro drift of the east; g is the acceleration of the gravity; L is the latitude; $\phi_{x}$, $\phi_{y}$, $\phi_{z}$ are the minimized alignment errors of pitch and roll and yaw, respectively [8,13].

### 2.2. Simulation

#### 2.2.1. Simulation Conditions

To facilitate the analysis, we firstly consider the alignment of static base conditions. The simulation conditions are shown in Table 2 and the sensors errors are shown in Table 3.

**Table 2:** Alignment under the two conditions.

Case 1	Static and with constant sensor errors
Case 2	Static and with constant and random sensor errors

**Table 3:** Sensors errors setting.

	Gyro Bias (°/h)		Accelerometer Bias (µg)	
	Constant	Random (White Noise)	Constant	Random (White Noise)
x-axis	0.04	0.04	50	50
y-axis	0.04	0.04	50	50
z-axis	0.04	0.04	50	50

In case 1, the simulation assumes that the ship is static, and the IMU instruments only have constant sensor errors. This case would not appear in practice, and it is just for theoretical analysis. The gravitational apparent motion in the inertial frame of the gravity vector is obtained in this case, which can be used as the reference standard in the improvement scheme. Therefore, in this case, there is no sway or instrument random error, and the gravity vector obtained by Equation (4) could be regarded as the theoretical value of the projection gravity vector in inertial frame. The simulation takes place in 118°E, 32°N with a sampling frequency of 200 Hz. The strapdown algorithm update cycle is 5 ms.

#### 2.2.2. Simulation Results

**Figure 5:**
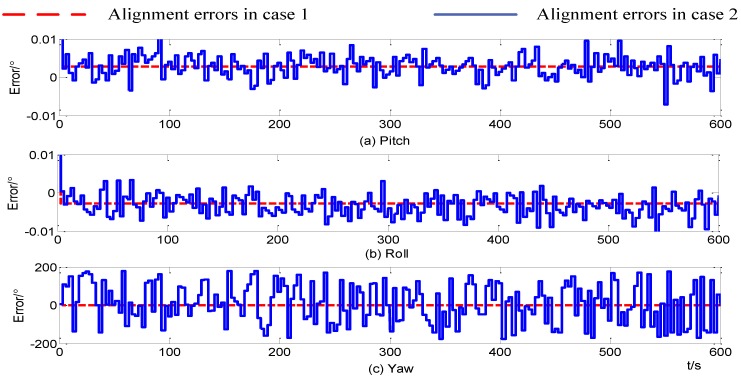
Curves of alignment errors.

In this paper, the simulation time period is 600 s and the alignment errors are shown in Figure 5 where only constant sensor errors in IMU can be seen, when the self-alignment for SINS based on three vectors of gravitational apparent motion in inertial frame can quickly complete the strapdown inertial navigation initial alignment. Alignment accuracy is equal to the theoretical value in Equation (10). When random sensor errors exist in IMU, the SINS alignment error increases, and an oscillation within 0.01° in the horizontal direction occurs; meanwhile, the yaw error is so severe that it is completely unavailable. Thus, the self-alignment for SINS based on three different vectors of gravitational apparent motion in inertial frame is heavily affected by the random sensor errors. Firstly, we analyze the reasons leading to the failure of the proposed alignment method.

#### 2.2.3. Reasons for the Alignment Failure

According to formulas from Equations (1)–(9), it can be concluded that the main factors that affect the alignment results are: (1) error of ${\hat{C}}_{b}^{i_{b_{0}}}$; (2) error of ${\hat{f}}^{i_{b_{0}}}$; (3) error of ${\vec{OO}}_{c}$; (4) error of $\hat{E}$.

##### (a) Error of ${\hat{C}}_{b}^{i_{b_{0}}}$

The errors of ${\hat{C}}_{b}^{i_{b_{0}}}$ are mainly composed of the calculation error and measurement error. However, the one-step upgrade showed that the calculation error is small enough and could be ignored. In [9] the authors pointed out that the gyros whose errors are equivalent to the gyros used in this paper can be introduced at a maximum of 0.0083° errors at 600 s in alignment because of the constant drift. The effect of random drift to the system only equals 4.1667 × 10^−7^°. Therefore, it is suggested that the error in ${\hat{C}}_{b}^{i_{b_{0}}}$ has no effect on the self-alignment for SINS based on three different vectors of gravitational apparent motion in the inertial frame.

##### (b) Error of ${\hat{f}}^{i_{b_{0}}}$

**Figure 6:**
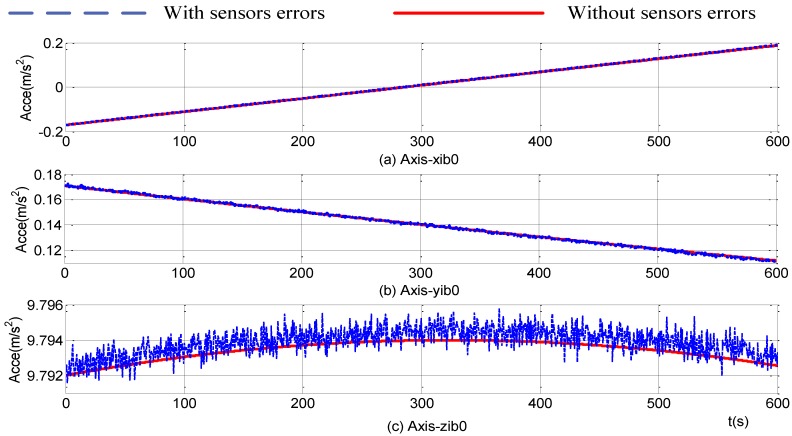
Projections of gravitational apparent motion in inertial frame.

From Equation (4), the projection of gravity vector in the apparent coordinate system is obtained by the coordinate transformation of the measurement of the accelerometers in the carrier coordinates. Therefore, with the analysis of error of ${\hat{C}}_{b}^{i_{b_{0}}}$, the projection accuracy of the gravity vector depends on the precision of the accelerometers. The projections of the gravity vector in the inertial frame under the situations of no instrument errors and both instrument constant and random errors are shown in Figure 6. The red solid line represents the theoretical value under no-error conditions. The blue dotted line shows the result when both instrument constant errors and random errors are present. It can be seen from Figure 6 that the random and the constant errors of accelerometers cause the random and constant errors of the gravity vector in the inertial frame, respectively.

##### (c) Error of ${\vec{OO}}_{c}$

From Equation (6), ${\vec{OO}}_{c}=\frac{\vec{AB}\times \vec{BC}}{\left\|\vec{AB}\times \vec{BC}\right\|}$, where $\vec{AB}={\hat{f}}^{i_{b_{0}}}(t_{B})-{\hat{f}}^{i_{b_{0}}}(t_{A})$ and $\vec{BC}={\hat{f}}^{i_{b_{0}}}(t_{C})-{\hat{f}}^{i_{b_{0}}}(t_{B})$. With the analysis in (b), it is known that ${\hat{f}}^{i_{b_{0}}}$ contains a large amount of random errors. If the intervals among *t*_A_, *t*_B_, *t*_C_ are too short, the existence of random errors may lead to the collinearity or dislocation of ${\hat{f}}^{i_{b_{0}}}(t_{A})$, ${\hat{f}}^{i_{b_{0}}}(t_{B})$ and ${\hat{f}}^{i_{b_{0}}}(t_{C})$, ${\hat{f}}^{i_{b_{0}}}(t_{B})$, which may contribute to the zero or reverse value of $\vec{AB}$, $\vec{BC}$. The vector ${\vec{OO}}_{c}$ obtained by Equation (6) will get a wrong or reverse value. Therefore, the random errors in ${\hat{f}}^{i_{b_{0}}}$ are the main reasons affecting ${\vec{OO}}_{c}$.

##### (d) Error of $\hat{E}$

From Equation (7), $\vec{E}=\frac{{\vec{OO}}_{c}\times \hat{U}}{\left\|{\vec{OO}}_{c}\times \hat{U}\right\|}$, among which $\hat{U}$ is directly obtained by ${\hat{f}}^{i_{b_{0}}}$. With the analysis in (c), ${\vec{OO}}_{c}$ is mainly affected by the random errors of ${\hat{f}}^{i_{b_{0}}}$. Therefore, the errors of $\hat{E}$ also result from ${\hat{f}}^{i_{b_{0}}}$.

Through the above analysis, the accuracy of the self-alignment for SINS based on three different vectors of gravitational apparent motion in the inertial frame is determined by ${\hat{f}}^{i_{b_{0}}}$. The random errors in ${\hat{f}}^{i_{b_{0}}}$ are the main reasons. However, the random errors of ${\hat{f}}^{i_{b_{0}}}$ are determined by the measurement of accelerometers in the carrier coordinates, so improvements are considered below which aim to solve the problems discussed previously.

## 3. Improved Methods

According to the analysis in Section 2, the self-alignment for SINS based on three different vectors of gravitational apparent motion in the inertial frame cannot be realized mainly because of the influence of the random measurement noise from the accelerometers. In order to reduce the influence of the random noise in IMU we introduce a Kalman filter which is more suitable for estimating the useful signal based on the knowledge about the system which reflects the behavior of the real world. The system behavior is described by the state-space representation where the uncertainties in the system could be considered as the measurement noise and process noise [26,27,28,29]. Inspired by these two ideals, a method based on an online parameters-adjusted Kalman filter is proposed to denoise the random noise of the IMU and a new reconstruction of the gravitational apparent motion vectors is also proposed.

### 3.1. A Dynamical IMU Data Denoising Technology Based on Kalman Filter

The Kalman filter which is used to estimate the states errors by the structure of the state space and *a priori* information of the process noise and the measurement noise is widely used in modern navigation systems. It can be used to effectively deal with the signals which are only disturbed by random noise or the disturbance can be treated as an independent variable of the system state variables [29]. The equivalent discrete system is as follows:

(11) $$X_{k+1}=\Phi \cdot X_{k}+B\cdot u_{k}+G\cdot w_{k},\;Z_{k+1}=H\cdot X_{k+1}+v_{k+1}$$

where, $X_{k}$ denotes the state vector at the time *k*; $Z_{k}$ denotes the system output (measured signal) at time *k*; $w_{k}$ denotes the process noise at the time *k*; $v_{k}$ denotes the measurement noise at the time *k*. F is the one step state transformation matrix; B is the input matrix (n × n). H is the measurement matrix (1 × n). $w_{k}$ and $v_{k+1}$ are the white noises with the expected value equal to zero.

(12) $$E(w_{k})=0,\;E(w_{k}\cdot w_{k}^{T})=Q_{k}\;E(v_{k})=\text{0},\;E(v_{k}\cdot v_{k}^{T})=R_{k}$$

where, $Q_{k}$ is the covariance matrix of the process noise $w_{k}$, $R_{k}$ is the covariance matrix of the measurement noise $v_{k}$.

In the Kalman filter, we ignore the control signal and consider the uncertainty part as the process noise and the measurement noise, then, the recursive equations of the Kalman filter are as follows:

(13) $$X_{k+1,k}=\Phi \cdot X_{k,k}$$

(14) $$P_{k+1,k}=\Phi \cdot P_{k,k}\cdot \Phi^{T}+G\cdot Q_{k}\cdot G^{T}$$

(15) $$K_{k+1}=P_{k+1,k}\cdot H^{T}\cdot {\left(H\cdot P_{k+1,k}\cdot H^{T}+R_{k+1}\right)}^{-1}$$

(16) $$X_{k+1,k+1}=X_{k+1,k}+K_{k+1}\cdot (Z_{k+1}-H\cdot X_{k+1,k})$$

(17) $$P_{k+1}=(I-K_{k+1}\cdot H)\cdot P_{k+1,k}$$

where $X_{k,k}$ is the state estimate at time *k*, $X_{k+1,k}$ is the one step predicted state, $P_{k,k}$ is the error covariance matrix at time *k*; $P_{k+1,k}$ is the predicted error covariance matrix; $K_{k+1}$ is the gain vector at time *k* + 1.

The first step in the design of the Kalman filter for this application is to build the state space models of the gyros and the accelerometers which can describe the different system behaviors in the real environment. Then, the measurements of the gyros and the accelerometers reflect the motion of the carriers, the detailed information of the disturbance and the control signal are difficult to determine, so the control signal is ignored and the disturbance is considered as the process noise and the measurement noise. In a low dynamic environment, little acceleration change is observed and it can be considered as a constant value within one sampling period. Then the state space model and the measurement model of the acceleration are [29]:

(18) $$\begin{array}{c} \dot{X}(t)=\frac{d}{dt}\left[\begin{array}{c} V\\ \dot{V} \end{array}\right]=\left[\begin{array}{cc} 0 & 1\\ 0 & 0 \end{array}\right]X(t)+\left[\begin{array}{cc} 1 & 0\\ 0 & 1 \end{array}\right]\cdot w(t)=F\cdot X(t)+B_{A}\cdot w(t)\\ Z(t)=[\begin{array}{cc} 0 & 1 \end{array}]\cdot X(t)+v(t)=C_{A}\cdot X(t)+v(t),\;X(t)=\left[\begin{array}{c} V\\ a \end{array}\right] \end{array}$$

where, V denotes the linear velocity of the carrier, a is the acceleration, Z(t) denotes the measurement of the acceleration at time *t*. v(t) denotes the measurement noise of the acceleration which is considered as white noise.

Similarly, it is assumed that the angular rate changes insignificantly and it could be regarded as a constant value within one sampling period as well. Then the state space model and the measurement model of the gyros are [29]:

(19) $$\begin{array}{c} \dot{X}(t)=\frac{d}{dt}\left[\begin{array}{c} \omega \\ \dot{\omega } \end{array}\right]=\left[\begin{array}{cc} 0 & 1\\ 0 & 0 \end{array}\right]X(t)+\left[\begin{array}{cc} 1 & 0\\ 0 & 1 \end{array}\right]\cdot w(t)=F\cdot X(t)+B_{G}\cdot w(t)\\ X(t)=[\begin{array}{cc} 1 & 0 \end{array}]\cdot X(t)+v(t)=C_{G}\cdot X(t)+v(t),\;X(t)=\left[\begin{array}{c} \omega \\ \varepsilon \end{array}\right] \end{array}$$

where, ω denotes the angular rate of the carrier, ε is the angular acceleration, Z(t) denotes the measurement of the gyros at time *t*. v(t) denotes the measurement noise of the gyros which is considered as white noise.

Equations (18) and (19) are the continuous system equations. Discretization processing is needed before the filtering iterative calculation according to the equations from Equations (13)–(17). The detailed approximate discretization steps are as follows:

(20) Φ≈I+T⋅F,   G≈T⋅B,   H=C

where *T* denotes the filter update cycle.

Although the state space model and measurement equation of the Kalman filter for one single input and one single output system is successfully built by Equations (18) and (19), whether the filter can work normally greatly depends on the initial parameters of the Kalman filter such as the variance matrix of the measurement noise $R_{k}(1\;\times \;1)$, the variance matrix of the process noise $Q_{k}(2\;\times \;2)$ and the one step prediction variance matrix $P_{0}(2\;\times \;2)$. In [29] the author has analyzed the influence of different ***Q**_k_*, $R_{k}$, $P_{0}$ on the Kalman filter in detail and an optimization approach for selecting the parameters of Kalman filter has been proposed, but these selected parameters do not work in a dynamic environment. In [30,31] the authors have proposed the adaptive moving average dual mode Kalman filter, which can adjust the filtering gain matrix ***K*** online.

According to the above analysis, the residual $\chi_{2}$ detection method is introduced in this paper to monitor the motion state of the carrier. When the value of the detection function is larger than the threshold $T_{D}$, it can be concluded that the carrier is in a rapidly changing state of motion. Then we should decrease the value of the matrix $R_{k}$ and increase the value of the matrix ***K*** to maintain the performance of the fast tracking to the carrier motion of the filter. When the value of the detection function is smaller than the threshold $T_{D}$, it can be concluded that the motion changes slowly or the carrier is in static conditions or in uniform motion in a straight line. At this time, we should enhance the denoising performance of the system by increasing the value of $R_{k}$ and reducing the value of ***K***. The detail instructions are described below:

The residual of the Kalman filter can be obtained from Equation (21):

(21) $$r_{k}=Z_{k}-H_{k}X_{k,k-1}$$

Then, the variance matrix of the residual is:

(22) $$A_{k}=H_{k}P_{k,k-1}H_{k}^{T}+R_{k}$$

After that, the detection function is:

(23) $$\gamma_{k}=r_{k}^{T}A_{k}^{-1}r_{k}$$

In order to test the online parameters-adjusted Kalman filter, one set of accelerometer data and gyro data are used and the filtering results of the online parameters-adjusted Kalman filter, finite impulse response filter (FIR) and wavelet filter are compared. Because the wavelet denoising technique is out of time-delay, here, the results of the wavelet filter just works as a reference point.

**Figure 7:**
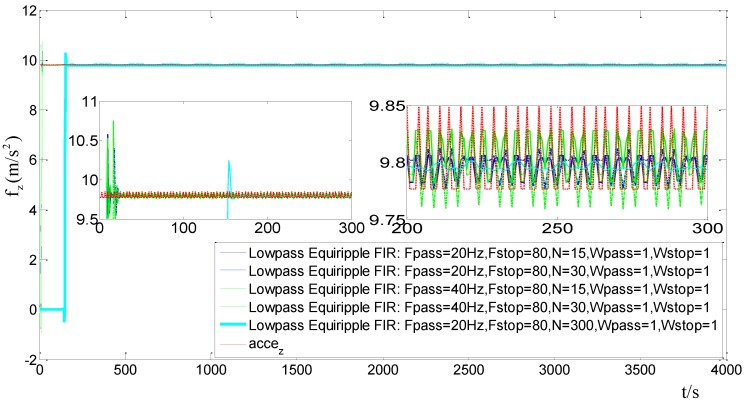
Filtering results with different filter parameters.

First, we should choose an optimal set of parameters for the FIR filter. In this paper, the MATLAB/Filter Design & Analysis Tool is used to design the FIR filter. The sampling frequency (Fs) equals 200 Hz. Figure 7 shows the results of FIR filters with different parameters when an accelerometer is in static. For the FIR filters, the transition-band cut-off frequency (Fpass) are set as 20 Hz and 40 Hz, respectively. The stop-band cut-off frequency (Fstop) is set to 80Hz, the filter order (N) are set to 15, 30 and 300, respectively. Both the transition-band weight value (Wpass) and the stop-band weight value (Wstop) are set to 1.

**Figure 8:**
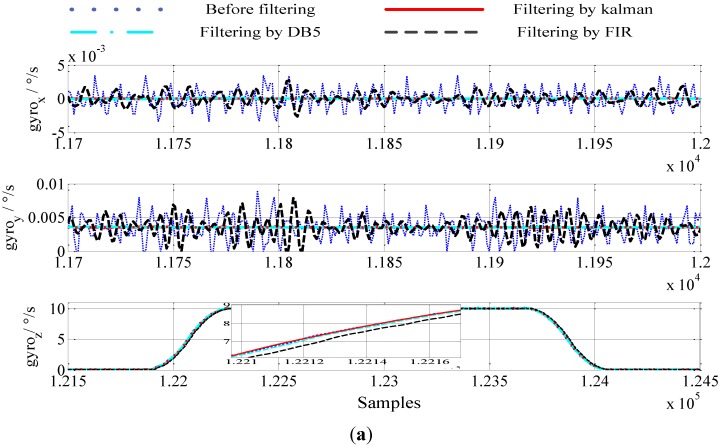

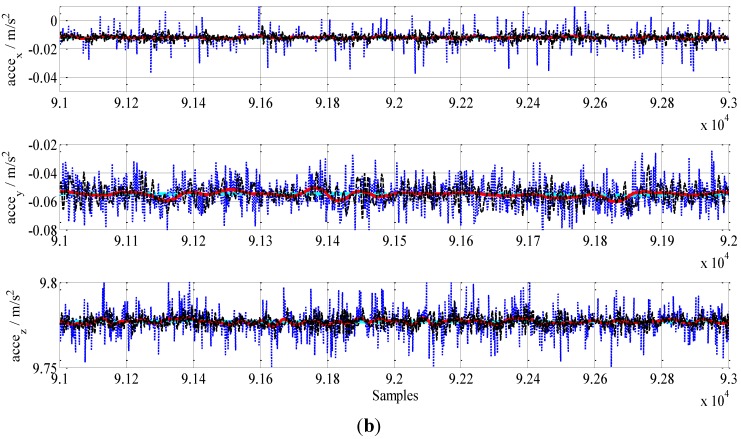
IMU dates in static (**a**) gyro dates (**b**) accelerometer dates.

**Figure 9:**
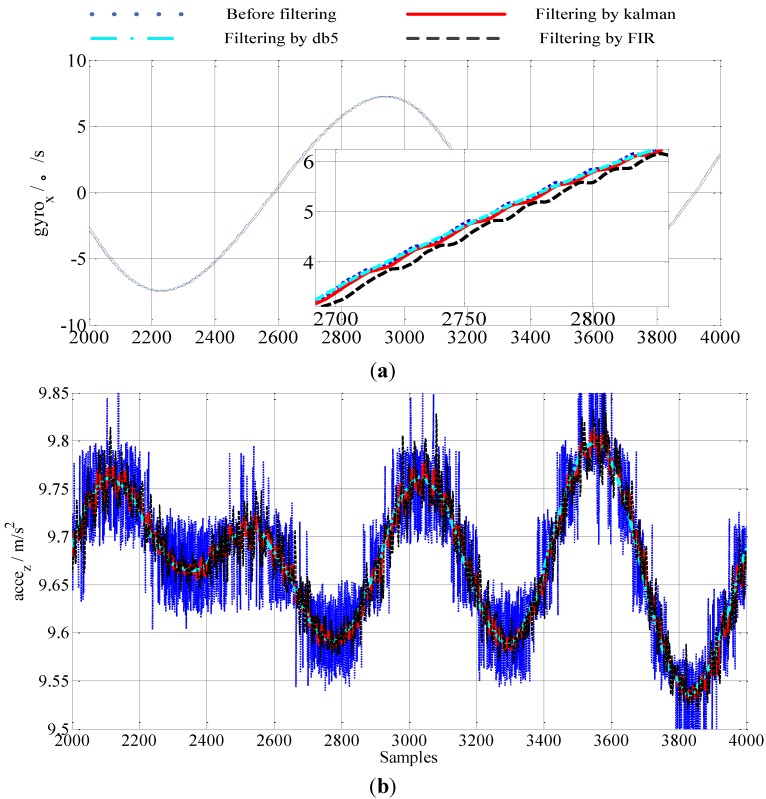
IMU data in swinging (**a**) gyro dates (**b**) accelerometer dates.

From Figure 7, it can be concluded that: (1) by maintaining the Fpass and Fstop constant, the high order of the filter results in a bigger time delay, but the filter results become better; (2) a low Fpass will result in better filtering results. Considering the time delay and the filter results, we choose an equiripple lowpass FIR filter whose Fpass equals 20 Hz, Fstop equals 80 Hz, and N equals 30. Figure 8 and Figure 9 are the results of the outputs of the gyros and accelerations before and after filtering. The abscissas of the figures denote the sampling point sequence.

In Figure 8, the IMU is kept static firstly. Then, the IMU rotates around axis *z* by 10°/s. Lastly, we keep the IMU static again. The blue dotted line denotes the output of the accelerometers and the gyros before filtering. The red solid line denotes the output of the accelerometers and the gyros after filtering by the online parameters-adjusted Kalman filter. The green dot dash line denotes the output of the accelerometers and the gyros after filtering by the wavelet filter. The wavelet function is the MATLAB wavelet function, db5, and the level of decomposition is 5. The black dashed line denotes the output of the accelerometers and the gyros after filtering by the FIR Filter. Comparing the results before and after filtering, it can be concluded that the measurement noise in the IMU can be effectively eliminated by the Kalman filter proposed in this paper and the wavelet. The filtering result of FIR is worse than the results of the other two filters, and there is an obvious time-delay when the IMU rotates.

In order to test the performance of the online parameters the adjusted Kalman filter proposed in this paper in a dynamic environment, the IMU outputs in a swinging case are used. Figure 9 shows the results before and after filtering the IMU outputs in the swinging case. From Figure 9, we can conclude that the filtering result of the wavelet filter is smoother than the other two filtering results. The filtering result of the online parameters-adjusted Kalman filter is better than the result of the FIR filter. From Figure 9 we can also conclude that the filtering results of the wavelet filter and the online parameters- adjusted Kalman filter can trace the motion of the carrier, while the filtering result has an obvious time-delay caused by the FIR filter in the swinging case. Although the delayed time of the FIR filter can be compensated accurately, the high order and the time-delay compensation would result in a great computational burden.

Because the parameters of the FIR are optimal when the IMU is in the static case, the filtering result of the FIR filter in the static case is better than that in the swinging case. Moreover, the measurement noise in the real environment is more complex, *i.e.*, when the ship is sailing on the sea, and the filtering result of the FIR filter would become worse because the filter parameters are set for a special situation, so comprehensively considering the filtering results in Figure 8 and Figure 9, we can conclude that the online parameters-adjusted Kalman filter is more suitable for a real-time system.

**Figure 10:**
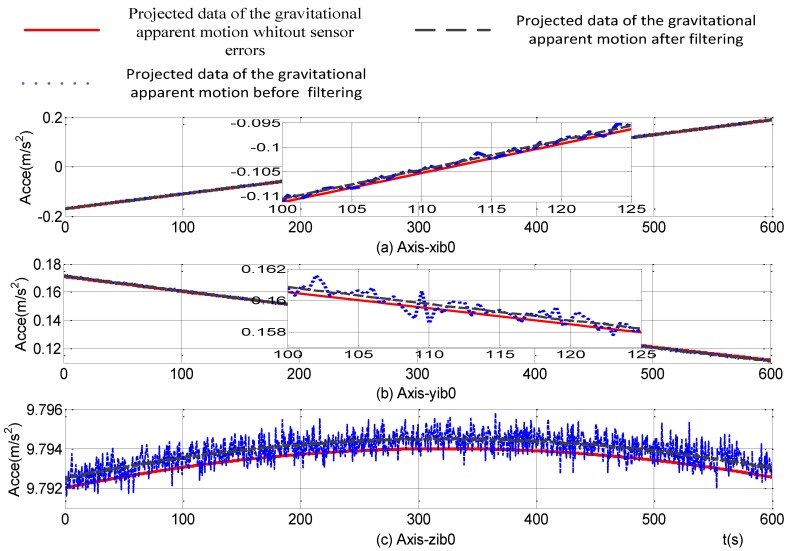
Projections of gravitational apparent motion in the inertial frame.

The introduction of online parameters-adjusted Kalman filter to the self-alignment of the SINS based on the three different vectors of gravitational apparent motion in the inertial frame and the projections of the gravity vectors measured by accelerometers are shown in Figure 10. From Figure 10, the projections of the gravity in the inertial frame, which are measured by accelerometers, are smoother and most of the random noise disturbance is eliminated. Compared with the theoretical gravity in the inertial frame without any IMU measurement errors, constant values exist because of the constant errors of the accelerometers.

### 3.2. Reconstruction for Apparent Motion

#### 3.2.1. Theoretical Expression of the Gravitational Apparent Motion in the Inertial Frame

In the inertial frame, the gravitational apparent motion is an ideal cone, and the cone parameters only depend on the latitude where the vehicle is located, but the specific expression of apparent motion should be studied in a fixed inertial frame, which means that the parameters and rules describing the theoretical apparent motion can be determined if and only if the inertial frame is selected.

According to Equation (4), the projection of theoretical apparent motion in inertial frame can be expressed as follows:

(24) $$f^{i_{b0}}(t)=C_{n}^{i_{b0}}(t)f^{n}=C_{n_{0}}^{i_{b0}}C_{e_{0}}^{n_{0}}C_{e}^{e_{0}}(t)C_{n}^{e}(t)f^{n}$$

where, $C_{n_{0}}^{i_{b_{0}}}$, $C_{e_{0}}^{n_{0}}$ are constant matrices, and $C_{n}^{e}(t)$ is also a constant value when there is no linear motion with the vehicle:

(25) $$C_{e}^{e_{0}}(t)={\left[\begin{array}{ccc} \cos[\omega_{ie}(t-t_{0})] & \sin[\omega_{ie}(t-t_{0})] & 0\\ -\sin[\omega_{ie}(t-t_{0})] & \cos[\omega_{ie}(t-t_{0})] & 0\\ 0 & 0 & 1 \end{array}\right]}^{T}$$

(26) $$C_{e_{0}}^{n_{0}}(t)=\left[\begin{array}{ccc} -\sin\lambda_{0} & \cos\lambda_{0} & 0\\ -\sin L_{0}\cos\lambda_{0} & -\sin L_{0}\sin\lambda_{0} & \cos L_{0}\\ \cos L_{0}\cos\lambda_{0} & \cos L_{0}\sin\lambda_{0} & \sin L_{0} \end{array}\right]$$

(27) $$f^{n}=\left[\begin{array}{c} 0\\ 0\\ -g \end{array}\right]$$

#### 3.2.2. Reconstruction for Apparent Motion

From Equation (24), $f^{i_{b0}}(t)=C_{n}^{i_{b0}}(t)f^{n}=C_{n_{0}}^{i_{b0}}C_{e_{0}}^{n_{0}}C_{e}^{e_{0}}(t)C_{n}^{e}(t)f^{n}$. If:

(28) $$f^{n_{0}}=C_{e_{0}}^{n_{0}}C_{e}^{e_{0}}(t)C_{n}^{e}(t)f^{n}$$

then:

(29) $$f^{i_{b0}}(t)=C_{n_{0}}^{i_{b0}}f^{n_{0}}(t)$$

where, $f^{n_{0}}(t)$ can be obtained from Equations (25)–(28).

If $f^{i_{b0}}(t)$ is accurate enough, we can freely record $f^{i_{b0}}(t)$ at two different moments, such as $f^{i_{b0}}(t_{A})$ and $f^{i_{b0}}(t_{B})$. Then the attitude matrix between the inertial frame and the initial navigation frame can be obtained by Equation (30):

(30) $$C_{i_{b0}}^{n_{0}}={\left[\begin{array}{c} {\text{[}f^{n_{0}}(t_{A})]}^{T}\\ {\text{[}f^{n_{0}}(t_{B})]}^{T}\\ {\text{[}f^{n_{0}}(t_{A})\times f^{n_{0}}(t_{B})]}^{T} \end{array}\right]}^{-1}\left[\begin{array}{c} {\text{[}f^{i_{b0}}(t_{A})]}^{T}\\ {\text{[}f^{i_{b0}}(t_{B})]}^{T}\\ {\text{[}f^{i_{b0}}(t_{A})\times f^{i_{b0}}(t_{B})]}^{T} \end{array}\right]$$

So the other projection of the gravity vector in the inertial frame at any time can be reconstructed by Equation (31):

(31) $$f^{i_{b0}}(t)=C_{n_{0}}^{i_{b0}}C_{e_{0}}^{n_{0}}C_{e}^{e_{0}}(t)C_{n}^{e}(t)f^{n}$$

Compared with the reconstruction method proposed in [9,22], the advantage of our proposed method is that it can be used in the alignment with the line motion carrier if the velocity of the vehicle is supported by other equipment.

### 3.3. Simulation

#### 3.3.1. Simulation Settings

In order to verify the correctness of the alignment scheme, simulations are conducted in two cases. The real environment of a ship, even under mooring conditions, is still a swinging condition, so the alignment conditions are set up in two ways as shown in Table 4. The constant errors and the random errors of IMU are shown in Table 5. In case 2, the ship is assumed to be in bad-moderate sea conditions and swings with the function $A*\sin(2\pi ft+\eta_{0})+\theta_{0}$, where A and f are the amplitude and frequency of swinging, while $\eta_{0}$ and $\theta_{0}$ are the initial phase and swinging center. The swinging parameters are shown in Table 6. To facilitate the following analysis, $\eta_{0}$ and $\theta_{0}$ are set as zeros. The latitude and longitude of the ship are set to 32°N and 118°E. The initial pith, roll and yaw errors are set to 0.4°, 0.4° and 5°, respectively.

**Table 4:** Alignment environment.

Case 1	Without swinging movement and with sensor errors
Case 2	With swinging movement and sensor errors

**Table 5:** Sensor errors.

	Gyro (°/h)		Accelerometer (µg)	
	Constant	Random (White Noise)	Constant	Random (White Noise)
x-axis	0.04	0.04	50	50
y-axis	0.04	0.04	50	50
z-axis	0.04	0.04	50	50

**Table 6:** Swing parameters.

	Pitch	Roll	Yaw
Amplitude (°)	6	8	4
Frequence (Hz)	0.12	0.15	0.1
Initial Phase (°)	0	0	0
Swinging center (°)	0	0	0

The initial Kalman filter parameters are set as follows:

$$X_{g0}={\left[\begin{array}{ccc} 0 & 0 & 0 \end{array}\right]}^{T},P_{g0}=diag\left[\begin{array}{ccc} 10 & 10 & 10 \end{array}\right],R_{g}=0.00001,Q_{g}=diag[\begin{array}{cc} 3\times 10^{-8} & 3\times 10^{-8} \end{array}]$$

$$X_{a0}={\left[\begin{array}{ccc} 0 & 0 & 0 \end{array}\right]}^{T},P_{a0}=diag\left[\begin{array}{ccc} 10 & 10 & 10 \end{array}\right],R_{a}=0.00001,Q_{a}=diag[\begin{array}{cc} 5\times 10^{-4} & 5\times 10^{-4} \end{array}]$$

When the value of the detection function is bigger than the threshold, $T_{D}$, the current filtering gain matrix ***K**_a_*, ***K**_g_* in the Kalman filter are multiplied by the coefficient which is equal to 2.1, 1.41, respectively, in this test. When the value of the detection function is smaller than the threshold $T_{D}$, then the current ***R**_g_* is multiplied by a coefficient which equals 1000 and the current $R_{a}$ is multiplied by a coefficient which equals 10,000.

#### 3.3.2. Simulation Results

**Figure 11:**
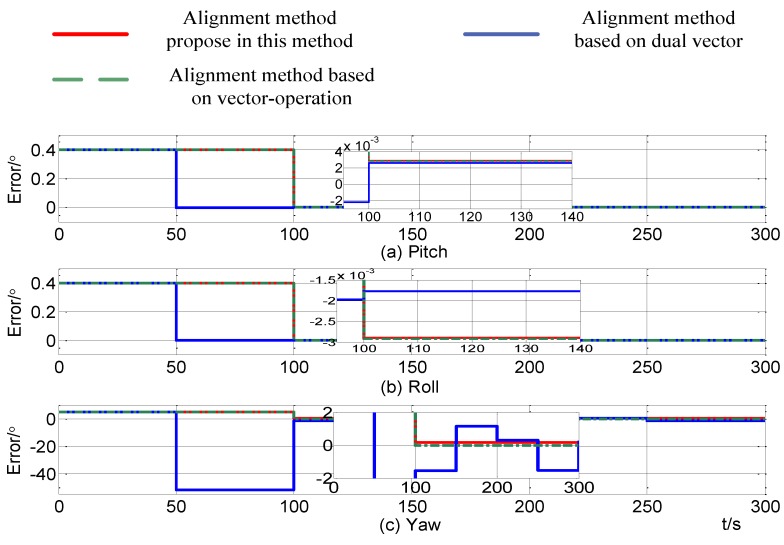
Alignment errors in case 1.

Figure 11 shows the alignment errors in case 1 which makes use of the improved self-alignment method proposed in this paper, the alignment method based on dual vectors and the method based on vector-operation. During the alignment process, the projection of the gravity at time stamps of 10 s, 50 s and 100 s are recorded in the alignment based on gravitational apparent motion and alignment based on vector-operation. The final alignment is at the 100 s time point. Because the alignment accuracy based on dual vectors cannot meet one full cycle of alignment requests within 100 s, two alignments based on dual vectors are needed. The first alignment completes at 50 s and the second alignment completes at 100 s. Figure 11 shows that all three alignment methods can meet the accuracy demands of the SINS initial alignment. The alignment time and alignment accuracy in horizontal mode in all three methods are nearly the same, but the alignment errors by the method based on three vectors proposed in this paper are more close to the theoretical values. In azimuth, the method based on three vectors is optimal. As a result the method based on vector-operation is suboptimal and the method based on dual vectors needs more time. Furthermore, Table 7 shows the alignment errors of the pitch, roll and yaw at 100 s obtained by these three methods.

**Table 7:** The alignment errors in case 1.

	The Theoretical Value	Method Based on Three Vectors	Method Based on Vector-Operation	Method Based on Dual Vectors
Pitch error (°)	0.0029	0.00288	0.00284	0.00265
Roll error (°)	−0.0029	−0.00285	−0.00293	−0.00177
Yaw error (°)	0.1802	0.1678	−0.00348	−1.552

**Figure 12:**
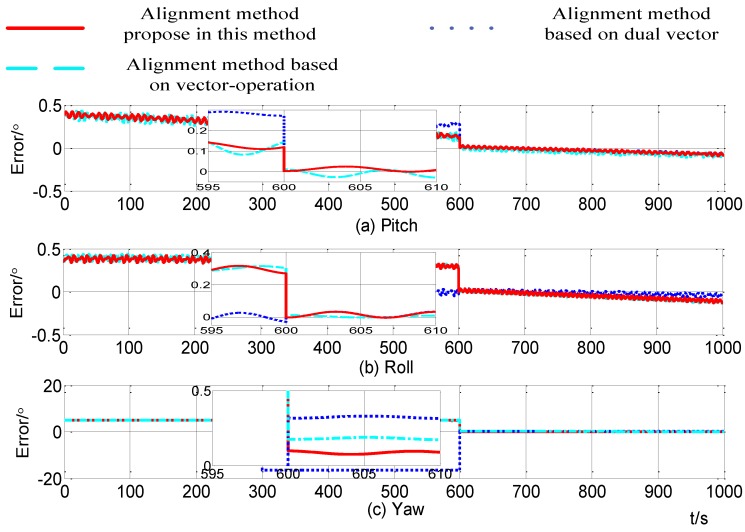
Alignment errors in case 2.

Figure 12 shows the alignment errors in case 2 by the improved self-alignment method proposed in this paper, the alignment method based on dual vectors and the method based on vector-operation. During the alignment process, the projection of the gravity at the time stamps of 10 s, 300 s and 600 s are recorded in the alignment based on gravitational apparent motion and alignment based on the vector-operation. The alignment is finished at the time 600 s and at that point the pitch, roll and yaw alignment errors obtained by these three methods are shown in Table 8.

**Table 8:** The alignment errors in case 1.

	The Theoretical Value	Method Based on Three Vectors	Method Based on Vector-Operation	Method Based on Dual Vectors
Pitch error (°)	0.0029	0.00285	0.00652	0.00289
Roll error (°)	−0.0029	−0.00289	0.00751	−0.00313
Yaw error (°)	0.1802	0.099	0.1765	0.318

## 4. Turntable Test and Vehicle Test

### 4.1. Test Configuration

#### 4.1.1. Turntable Test Configuration

In the turntable test, the attitude angle information of the turntable can be used as the attitude reference which can be transferred by the serial communication port and synchronized with the external clock signal. Then the inner, intermediate and outer frames can be used to simulate the roll, pitch and yaw of the ship, respectively. The motion state of the turntable is shown in Table 9 and Table 10. A prototype strapdown inertial navigation system whose sensors are three fiber optic gyroscopes and three quartz accelerometers is used in this experiment. The precision of the sensors is shown in Table 11.

**Table 9:** Case 1 motion state of the turntable.

	Inner	Intermediate	Outer
Position (°)	21	30	39

**Table 10:** Case 2 motion state of the turntable.

	Pitch	Roll	Yaw
Amplitude (°)	8	10	6
Frequence (Hz)	0.12	0.15	0.1
Swing center (°)	0	0	0

**Table 11:** The sensors precision of the strapdown inertial navigation system.

Gyro Bias		Accelerometer Bias	
Constant	Random (white noise)	Constant	Random (white noise)
＜0.01 ^○^/h	＜0.01 ^○^/h	±5 × 10^−5^ g	＜5 × 10^−5^ g

**Figure 13:**
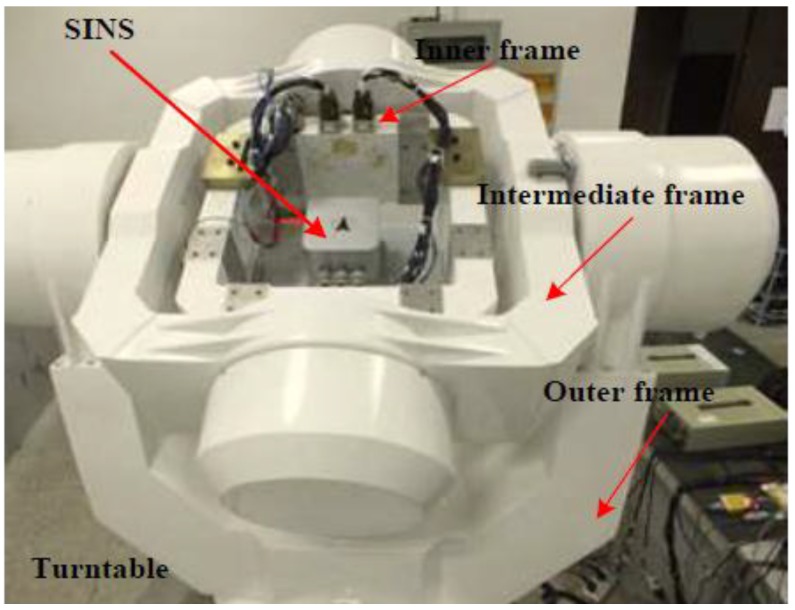
The turntable and the IMU.

**Figure 14:**
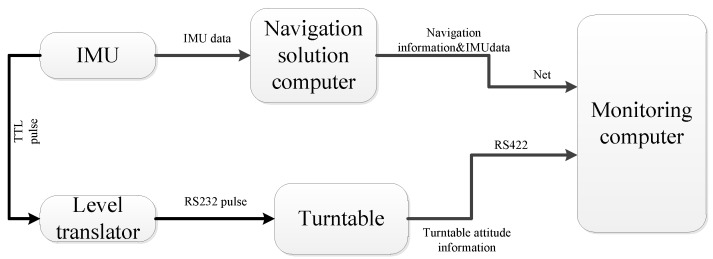
Construction of the navigation system.

The IMU is installed in the turntable as shown in Figure 13 with the axes of the x, y and z coinciding with the axes of intermediate, inner and outer frames, respectively. The sensors zeros bias, scale coefficients, coupling coefficients and installation errors can be determined and compensated following the method described in [32]. Furthermore, the data from IMU and turntable are updated at a rate of 200 Hz. The strapdown inertial navigation system and experimental environment are shown in Figure 14.

#### 4.1.2. Vehicle Test Configuration

**Figure 15:**
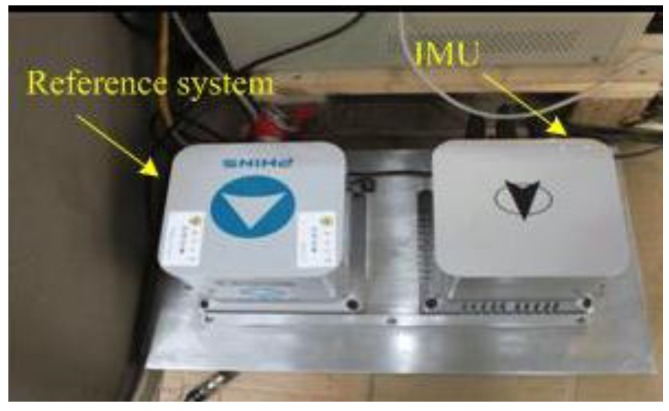
Installation diagram.

**Figure 16:**
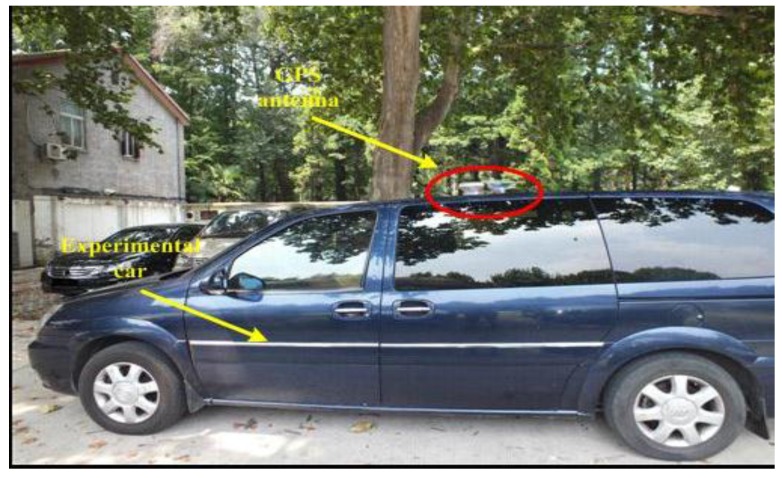
Experimental car.

The vehicle test was conducted in a car which was used to simulate the maneuvers of an underwater vehicle. The parameters of gyros and the accelerometers are shown in Table 11. The reference navigation data come from the loose couple of PHINS developed by the French firm IXBLUE and the FlexPark6 GNSS receiver developed by the firm NovAtel (Calgary, Alberta, Canada). The performance of the PHINS in GPS aided mode is as follows: both pitch and roll errors are less than 0.01°, heading error is less than 0.01°sec*L* (*L* is the location latitude) [33]. PHINS and inertial measurement unit are fixed on the same mounting plate shown in Figure 15. Figure 16 is the navigation experimental car with a red circle to mark the GNSS receiver antenna. The car is kept static but the engine operates normally during the alignment process. The personnel is allowed to move in the car.

### 4.2. Alignment Results

#### 4.2.1. Turntable Test Result

**Figure 17:**
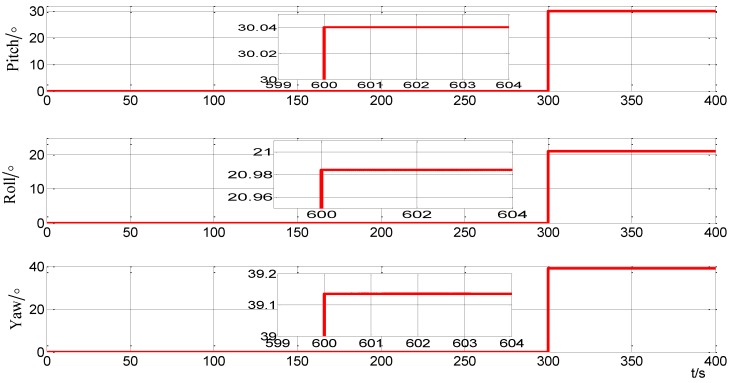
Curves of alignment for the turntable test in static.

In order to verify the performance of the alignment scheme in an arbitrary position, the turntable is set as in Table 9. Because the measurement noise is more complicated, in order to reduce the influence of the complicated measurement noise and to obtain a more accurate $C_{i_{b_{0}}}^{n_{0}}$, the alignment time is set to 300 s. The system recorded the projection of the gravity at the time stamps of 10 s, 150 s and 300 s, where 300 s is set as a final alignment measurement. Figure 17 shows the alignment results by using the improved self-alignment for SINS based on the three vectors of gravitational apparent motion in the inertial frame proposed in this paper. The alignment errors are shown in Table 12.

**Figure 18:**
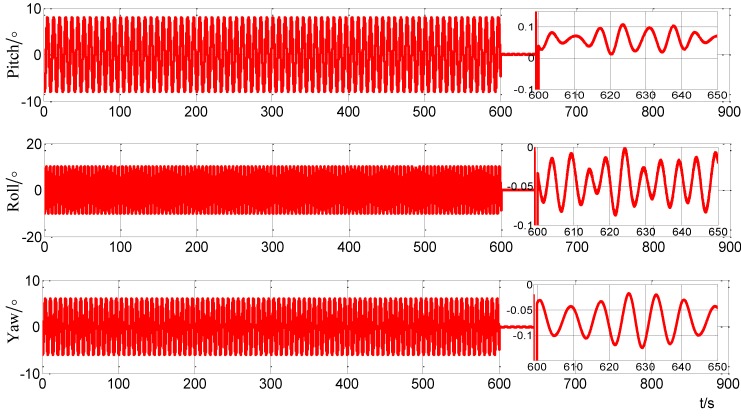
Curves of alignment for the turntable test in swing.

In order to verify the performance of the alignment scheme in swinging state, the turntable is set as described in Table 10. Due to the swinging case, the coupling between the IMU is enhanced. In order to reduce the influence of the complicated measurement noise and to obtain a more accurate $C_{i_{b_{0}}}^{n_{0}}$, the alignment time is extended and set at 600 s. The system recorded the projection of the gravity at 10 s, 300 s and 600 s. Meanwhile, the alignment is finished at the time 600 s. Figure 18 shows the alignment results by using the improved self-alignment for SINS based on the three vectors of gravitational apparent motion in the inertial frame proposed in this paper. The alignment errors are shown in Table 12.

**Table 12:** The alignment errors in turntable test.

	Case 1	Case 2
Pitch error (°)	0.0395	0.04
Roll error (°)	0.0211	−0.033
Yaw error (°)	0.14	−0.045

#### 4.2.2. Vehicle Test Result

**Figure 19:**
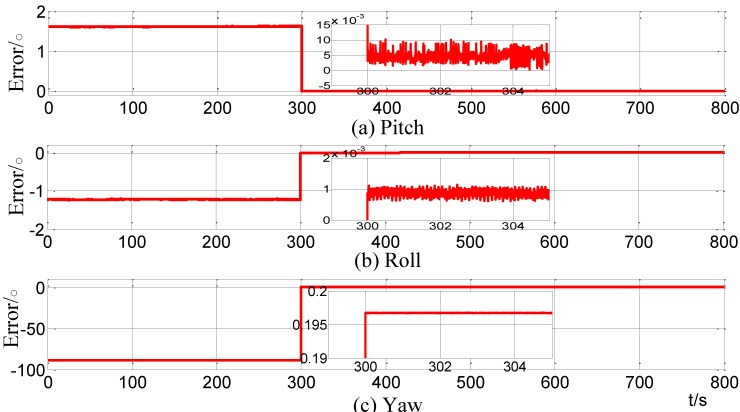
Curves of alignment errors for the vehicle test.

The alignment errors of the vehicle test, which take the PHINS attitude as the reference, are shown is Figure 19. When the coarse alignment completes at 300 s, the pitch, roll and yaw errors are 0.00305°, 0.000872° and 0.1957°, respectively.

## 5. Conclusions

In this paper, an improved self-alignment for SINS based on tracing the apparent motion in an inertial frame is designed. In this proposed method, the gravitational apparent motion vectors at three different moments are selected to construct the attitude matrix between the inertial body frame and the current navigation frame with vector-operations. Taking advantage of the gyro outputs, the attitude matrix between the current body frame and inertial body frame can be acquired. Thus, attitude between current body frame and navigation frame can be solved through the multiplication of the above shown matrices.

Simulation and analysis indicate that the proposed method easily suffers from random noise contained in the accelerometer measurements which are used to construct the apparent motion directly from the acceleration input. To solve this problem, a sensor data denoising method by an online parameters-adjusted Kalman filter is designed and described in detail in this paper and a new reconstruction algorithm for apparent motion is devised.

Simulation, turntable tests and vehicle tests show that the alignment method can meet the initial alignment needs for SINS in static and swinging conditions. The accuracy can reach or approach the theoretical values determined by sensor precision under static or swinging conditions.
